# Concomitant medications in patients with metastatic urothelial carcinoma receiving enfortumab vedotin: real-world data from the ARON-2^EV^ study

**DOI:** 10.1007/s10585-025-10335-4

**Published:** 2025-02-20

**Authors:** Ondřej Fiala, Sebastiano Buti, Kazutoshi Fujita, Alfonso Gómez de Liaño, Wataru Fukuokaya, Takahiro Kimura, Takafumi Yanagisawa, Patrizia Giannatempo, Martin Angel, Alessia Mennitto, Javier Molina-Cerrillo, Maria T. Bourlon, Andrey Soares, Hideki Takeshita, Fabio Calabrò, Cinzia Ortega, Jakub Kucharz, Michele Milella, Emmanuel Seront, Se Hoon Park, Deniz Tural, Giovanni Benedetti, Yüksel Ürün, Nicola Battelli, Bohuslav Melichar, Alexandr Poprach, Tomas Buchler, Jindřich Kopecký, Vincenza Conteduca, Fernando Sabino Marques Monteiro, Francesco Massari, Shilpa Gupta, Matteo Santoni

**Affiliations:** 1 Department of Oncology and Radiotherapeutics, Faculty of Medicine and University Hospital in Pilsen, Charles University Prague, Alej Svobody 80, 304 60 Pilsen, Czech Republic; 2 Biomedical Center, Faculty of Medicine in Pilsen, Charles University, Pilsen, Czech Republic; 3 Medical Oncology Unit, University Hospital of Parma, Parma, Italy; 4 Department of Medicine and Surgery, University of Parma, Parma, Italy; 5 Department of Urology, Kindai University Faculty of Medicine, Osaka, Japan; 6 Department of Medical Oncology, Complejo Hospitalario Universitario Insular-Materno Infantil, Las Palmas, Spain; 7 Department of Urology, The Jikei University School of Medicine, 3-19-18 Nishi-Shimbashi, Minato-Ku, Tokyo, 105-8471 Japan; 8 Medical Oncology Department, Fondazione IRCCS Istituto Nazionale Dei Tumori, Via Giacomo Venezian 1, Milan, Italy; 9 Clinical Oncology, Genitourinary Oncology Unit, Alexander Fleming Institute, Buenos Aires, Argentina; 10 Department of Medical Oncology, Azienda Ospedaliera Universitaria “Maggiore Della Carit”, Novara, Italy; 11 Department of Medical Oncology, Hospital Ramón y Cajal, Madrid, Spain; 12 Department of Hemato-Oncology, Instituto Nacional de Ciencias Medicas y Nutricion Salvador Zubiran, Escuela de Medicina, Mexico-Universidad Panamericana, Mexico City, Mexico; 13 Hospital Israelita Albert Einstein, São Paulo, SP Brazil; 14 Latin American Cooperative Oncology Group-LACOG, Porto Alegre, Brazil; 15 Department of Urology, Saitama Medical Center, Saitama Medical University, Saitama, Japan; 16 Medical Oncology 1-IRCCS Regina Elena National Cancer Institute, Rome, Italy; 17 Dipartimento di Oncologia, Ospedale Michele E Pietro Ferrero-Verduno (CN) ASLCN2 Alba E, Bra, Italy; 18 Department of Uro-Oncology, Maria Sklodowska-Curie National Research Institute of Oncology Warsaw, Warsaw, Poland; 19 Section of Innovation Biomedicine-Oncology Area, Department of Engineering for Innovation Medicine (DIMI), University and Hospital Trust (AOUI) of Verona, 37134 Verona, Italy; 20 Department of Medical Oncology, Cliniques Universitaires Saint-Luc, Brussels, Belgium; 21 Samsung Medical Center, Sungkyunkwan University School of Medicine, Seoul, Korea; 22 Department of Medical Oncology, Koc University Medical Faculty, Istanbul, Türkiye; 23 U.O. Oncologia, Ospedale Di Civitanova Marche, Civitanova Marche, Italy; 24 Department of Medical Oncology, Ankara University Faculty of Medicine, 06620 Ankara, Türkiye; 25 Medical Oncology Unit, Macerata Hospital, Macerata, Italy; 26 Department of Oncology, Faculty of Medicine and Dentistry, Palacký University, Olomouc, Czech Republic; 27 Department of Comprehensive Cancer Care, Masaryk Memorial Cancer Institute, 656 53 Brno, Czech Republic; 28 Department of Comprehensive Cancer Care, Faculty of Medicine, Masaryk University, 625 00 Brno, Czech Republic; 29 Department of Oncology, Second Faculty of Medicine, Charles University and Motol University Hospital, V Úvalu 84, 150 06 Prague, Czech Republic; 30 Department of Oncology, University Hospital in Hradec Králové, Sokolská 581, 50005 Hradec Králové, Czech Republic; 31 Unit of Medical Oncology and Biomolecular Therapy, Department of Medical and Surgical Sciences, University of Foggia, Policlinico Riuniti, Foggia, Italy; 32 Hospital Sírio-Libanês, Brasília, DF Brazil; 33 Medical Oncology, IRCCS Azienda Ospedaliero-Universitaria Di Bologna, Bologna, Italy; 34 Department of Medical and Surgical Sciences (DIMEC), University of Bologna, Bologna, Italy; 35 Taussig Cancer Institute, Cleveland Clinic, Cleveland, OH USA

**Keywords:** Urothelial cancer, Enfortumab vedotin, Concomitant medication, Antibiotics, Corticosteroids, Concomitant drug score, ARON-2^EV^ study, NCT05290038

## Abstract

Patients with metastatic urothelial carcinoma (mUC) are typically elderly and often have other comorbidities that require the use of concomitant medications. In our study we evaluated the association of concomitant use of antibiotics (ATBs), proton pump inhibitors (PPIs), corticosteroids, statins, metformin and insulin with patient outcomes and we validated the prognostic role of a concomitant drug score in mUC patients treated with enfortumab vedotin (EV) monotherapy. Data from 436 patients enrolled in the ARON-2^EV^ retrospective study were analyzed according to the concomitant medications used at baseline. Finally, the patients were stratified into three risk groups according to the concomitant drug score based on ATBs, corticosteroids and PPIs. Statistical analysis involved Fisher exact test, Kaplan–Meier method, log-rank test, and univariate/multivariate Cox proportional hazard regression models. Inferior survival outcomes were observed in ATB users compared to non-users (OS: 7.3 months, 95%CI 5.0 − 12.3 vs 13.7 months, 95%CI 12.2 − 47.3, p = 0.001; PFS: 5.1 months 95%CI 3.3 − 17.7 vs 8.3 months, 95%CI 7.1 − 47.3, p = 0.001) and also in corticosteroid users compared to non-users (OS: 8.4 months, 95%CI 6.6 − 10.0 vs 14.2 months, 95%CI 12.7 − 47.3, p < 0.001; PFS: 6.0 months 95%CI 4.6 − 7.9 vs 8.9 months, 95%CI 7.2 − 47.3, p = 0.004). In the Cox multivariate analysis, the concomitant drug score was a significant factor predicting both OS (HR = 1.32 [95% CI 1.03 − 1.68], p = 0.026) and PFS (HR = 1.23 [95% CI 1.01 − 1.51], p = 0.044). Our findings suggest detrimental impact of concomitant use of ATBs and corticosteroids on survival outcomes and the prognostic utility of the concomitant drug score in previously treated mUC patients receiving EV.

## Introduction

Urothelial carcinoma (UC) affects the urinary tract, including the bladder, ureters, and renal pelvis. Predominantly conventional UC is the most common histologic type of tumor of the upper and lower urinary tract, accounting for approximately 90% of all cases [1]. The landscape of systemic therapies for patients with locally advanced or metastatic UC (la/mUC) has evolved with the introduction of new effective anticancer agents. The most important novel agents that have established themselves in the treatment of la/mUC in recent years are an antibody–drug conjugate (ADC), enfortumab vedotin (EV), and a number of immune checkpoint inhibitors (ICIs) targeting the programmed cell death protein/ligand-1 (PD-1/PD-L1) pathway, including pembrolizumab, avelumab, atezolizumab and nivolumab. EV is an antibody targeting nectin-4 linked to the microtubule disrupting agent monomethyl auristatin E (MMAE) [2]. EV monotherapy proved efficacy for patients with la/mUC previously treated with platinum-based chemotherapy followed by PD-1/PD-L1 inhibitors or those cisplatin-ineligible who have previously received one or more prior lines of systemic therapy [3, 4]. The combination of EV and pembrolizumab has currently become the preferred first-line treatment for la/mUC [5].

Patients with la/mUC are typically elderly. They often have other relevant comorbidities, which necessitates the use of a significant number of concomitant medications. In recent years, there has been a growing interest in the impact of commonly used drugs on cancer patient outcomes. A number of studies have indicated that concomitant medications may influence the efficacy and survival outcomes of systemic anticancer therapies in patients with a range of malignancies. The most common drugs of interest include antibiotics (ATBs), proton pump inhibitors (PPIs), corticosteroids, statins, and antidiabetics. These drugs are widely used in the general population. In cancer patients specifically, some of these agents are used to alleviate the side effects associated with anticancer therapy. In particular, ATBs, PPIs and corticosteroids have recently attracted attention because of their potentially detrimental impact on the efficacy of ICIs, which have been reported in numerous studies [6–14]. Using these three drug types, a concomitant drug score has been developed to predict survival outcomes in cancer patients receiving ICIs and its feasibility has been validated in several malignancies, including la/mUC [15–19]. To the best of our knowledge, there are currently no data available on the potential role of concomitant medications in mUC patients treated with EV.

The ARON-2^EV^ study (NCT05290038) was designed to collect real-world data worldwide on the use of EV for the treatment of patients with mUC. The objective of the present analysis was twofold: firstly, to evaluate the association of concomitant use of ATBs, PPIs, corticosteroids, statins, metformin and insulin with patient outcomes; and secondly, to validate the prognostic role of a previously proposed concomitant drug score in the ARON-2^EV^ study population.

## Patients and methods

### Study design, patients and objectives

We retrospectively analyzed the anonymized clinical records of patients with histologically confirmed mUC who were treated with EV after frontline platinum-based chemotherapy and second-line pembrolizumab or avelumab maintenance. The patients were treated between January 1st, 2022 and August 30th, 2024. The clinical data including concomitant medication were obtained from hospital information systems submitted by centers participating in the ARON2^EV^ study, which involved 58 oncological centers from 15 countries. The principal objective of this sub-analysis of the ARON2^EV^ study was to assess the relationship between the concomitant administration of selected medications within the first month of EV treatment and the outcomes of progression-free survival (PFS), overall survival (OS), and overall response rate (ORR). The concomitant medications evaluated included ATBs, PPIs, corticosteroids, statins, metformin, and insulin. The secondary objective was to validate the prognostic ability of the previously proposed concomitant drug score [15–19]. These medications were administered at usual doses under the supervision of the patients’ healthcare providers.

The study protocol was approved on September 28, 2023, by the Ethical Committee of the coordinating center (Marche Region, Italy-No. 2022 39/7875) and by the Institutional Review Boards of participating centers. The study was conducted according to Good Clinical Practice (GCP) and International Ethical Guidelines for Biomedical Research, and the protocol has been designed with the ethical principles laid down in the Declaration of Helsinki on human experimentation. The Informed consent with subsequent analysis of the clinical data was obtained from all the included patients.

### Treatment and outcome assessment

EV was administered intravenously as a single agent in the standard approved schedule (1.25 mg/kg given on Days 1, 8, and 15 of each 28-day cycle). The treatment was continued until disease progression, unacceptable toxicity, or patient refusal. Physical examination, laboratory tests and computed tomography (CT) scans were performed at baseline and every 2–4 months thereafter, according to local practice, or when disease progression was clinically suspected.

PFS was defined as the time from the start of EV treatment to progression or death from any cause, whichever occurred first. OS was defined as the time from the start of EV treatment until death or lost at follow-up. We considered as censored those patients without progression or death at the last follow-up. The objective response to EV was assessed according to RECIST 1.1 and data on complete (CR) or partial responses (PR), stable disease (SD) or progressive disease (PD) were collected and analyzed [20]. ORR was defined as the proportion of patients who achieved CR or PR.

### Calculation of concomitant drug score

The calculation method of the concomitant drug score has been described in detail previously [17–22]. Briefly, the following drugs used within one month before EV initiation were scored: ATBs (score = 1), PPIs (score = 1), and corticosteroids (score = 2). The total score was calculated by adding the component scores. Finally, the patients were stratified into the following three risk groups: good risk (total score = 0), intermediate risk (total score = 1–2), and poor risk (total score = 3–4).

### Statistical analysis

PFS and OS were estimated by employing the Kaplan–Meier method with Rothman’s 95% confidence intervals (CI) and compared using the log-rank test. The median follow-up was calculated with the Kaplan–Meier method. Univariate and multivariate analyses were performed using Cox proportional hazards models. In the multivariate model, factors with a significant impact on survival in the univariate model were evaluated. The chi-square test was utilized to assess potential differences between evaluated medication users and non-users in terms of patient characteristics and ORR. Significance levels were set at a 0.05 value and all *p* values were two-sided. The statistical analysis was performed by using MedCalc version 19.6.4 (MedCalc Software, Broekstraat 52, 9030 Mariakerke, Belgium).

## Results

### Patient characteristics

Four hundred and thirty-six patients were included in the present analysis. The median follow-up time was 12.8 months (95%CI 9.0−65.8); 328 patients (75%) were males (Table 1). Median age was 70 years (range 37−90). Tumor histology was pure UC in 367 patients (84%). Lymph node and lung metastases were identified in 76% and 50% of patients, respectively. In 229 patients (53%), EV was ongoing at the time of data cut-off; 158 patients (36%) had died at time of data cut-off; 182 patients (42%) received EV following progression during maintenance therapy with avelumab and 254 (58%) after progression during pembrolizumab. Seventy-two patients who progressed during EV therapy received successive treatments. Concomitant use of antibiotics, PPIs, corticosteroids, statins, metformin and insulin was reported in 41 (9%), 223 (51%), 101 (23%), 101 (23%), 53 (12%) and 29 patients (7%), respectively (Table 1). Patients’ characteristics are summarized in Table 1. The use of statins was significantly more frequent in patients aged < 70 y, while concomitant antibiotics and/or corticosteroids were more frequently administered in patients with ECOG-PS ≥ 2 (Table 1).

**Table 1:** Clinico-pathological characteristics of patients treated with enfortumab eedotin stratified by concomitant medications

Patients	Overall	Proton pump inhibitor users 223 (%)	Proton pump inhibitor non-users 213 (%)	*p*	Antibiotic users	Antibiotic non-users	*p*	Corticosteroid users	Corticosteroid non-users	*p*
	436 (%)				41 (%)	395 (%)		101 (%)	335 (%)	
*Gender*				0.747			0.522			0.747
Male	328 (75)	170 (76)	158 (74)		29 (71)	299 (76)		75 (74)	253 (76)	
Female	108 (25)	53 (24)	55 (26)		12 (29)	96 (24)		26 (26)	82 (24)	
Age ≥ 70y	204 (47)	106 (48)	98 (46)	0.887	6 (15)	198 (50)	** < 0.001**	23 (23)	181 (54)	** < 0.001**
ECOG-PS ≥ 2	71 (16)	43 (19)	28 (13)	0.335	16 (39)	55 (14)	** < 0.001**	34 (34)	37 (11)	** < 0.001**
Current or former smokers	278 (64)	145 (65)	133 (62)	0.769	27 (66)	251 (64)	0.882	59 (58)	219 (65)	0.383
*Primary tumor location*				0.535			0.346			0.342
Upper urinary tract	129 (30)	61 (27)	68 (32)		10 (24)	119 (30)		24 (24)	105 (31)	
Lower urinary tract	307 (70)	162 (73)	145 (68)		31 (76)	276 (70)		77 (76)	230 (69)	
*Tumor histology*				1.000			1.000			1.000
Pure urothelial carcinoma	367 (84)	187 (84)	180 (85)		35 (85)	332 (84)		85 (84)	282 (84)	
Minor or mixed variants	69 (16)	36 (16)	33 (15)		6 (15)	63 (16)		16 (16)	53 (16)	
Synchronous metastastic disease	158 (36)	72 (32)	86 (40)	0.302	15 (37)	143 (36)	1.000	27 (27)	131 (39)	0.074
*Common sites of metastasis*										
Lymph nodes (non-regional)	338 (76)	173 (78)	165 (77)	1.000	29 (71)	309 (78)	0.330	80 (79)	258 (77)	0.865
Lung	218 (50)	115 (52)	103 (48)	0.671	21 (51)	197 (50)	1.000	46 (46)	172 (51)	0.572
Bone	154 (35)	82 (37)	72 (34)	0.768	16 (39)	138 (35)	0.660	47 (47)	107 (32)	**0.043**
Liver	148 (34)	84 (38)	64 (30)	0.296	21 (51)	127 (32)	**0.010**	39 (39)	109 (33)	0.462
Brain	15 (3)	11 (5)	4 (2)	0.282	4 (10)	11 (3)	0.082	9 (9)	6 (2)	0.058
EV after progression to avelumab	182 (42)	89 (40)	93 (44)	0.667	10 (24)	172 (44)	**0.004**	36 (36)	146 (44)	0.312
EV after progression to pembrolizumab	254 (58)	134 (60)	120 (56)	0.667	31 (76)	223 (56)	**0.004**	65 (64)	189 (56)	0.312

Statistically significant differences are in bold

*ECOG-PS* Eastern cooperative oncology group-performance status, *UC* Urothelial carcinoma, *EV* Enfortumab vedotin

### Survival outcomes

**Fig. 1:**
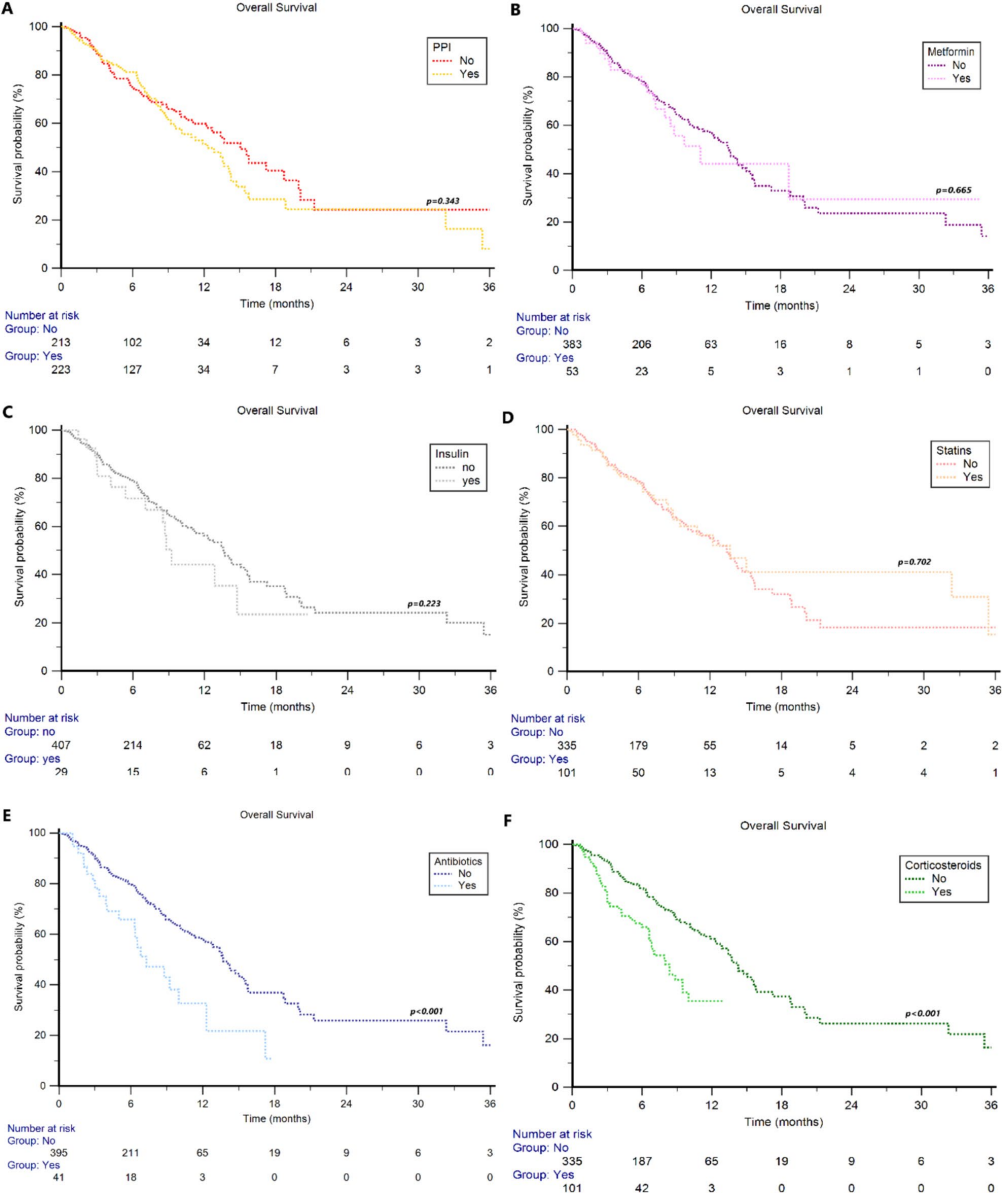
Overall Survival in patients treated with enfortumab vedotin (EV) stratified by concomitant medications

**Fig. 2:**
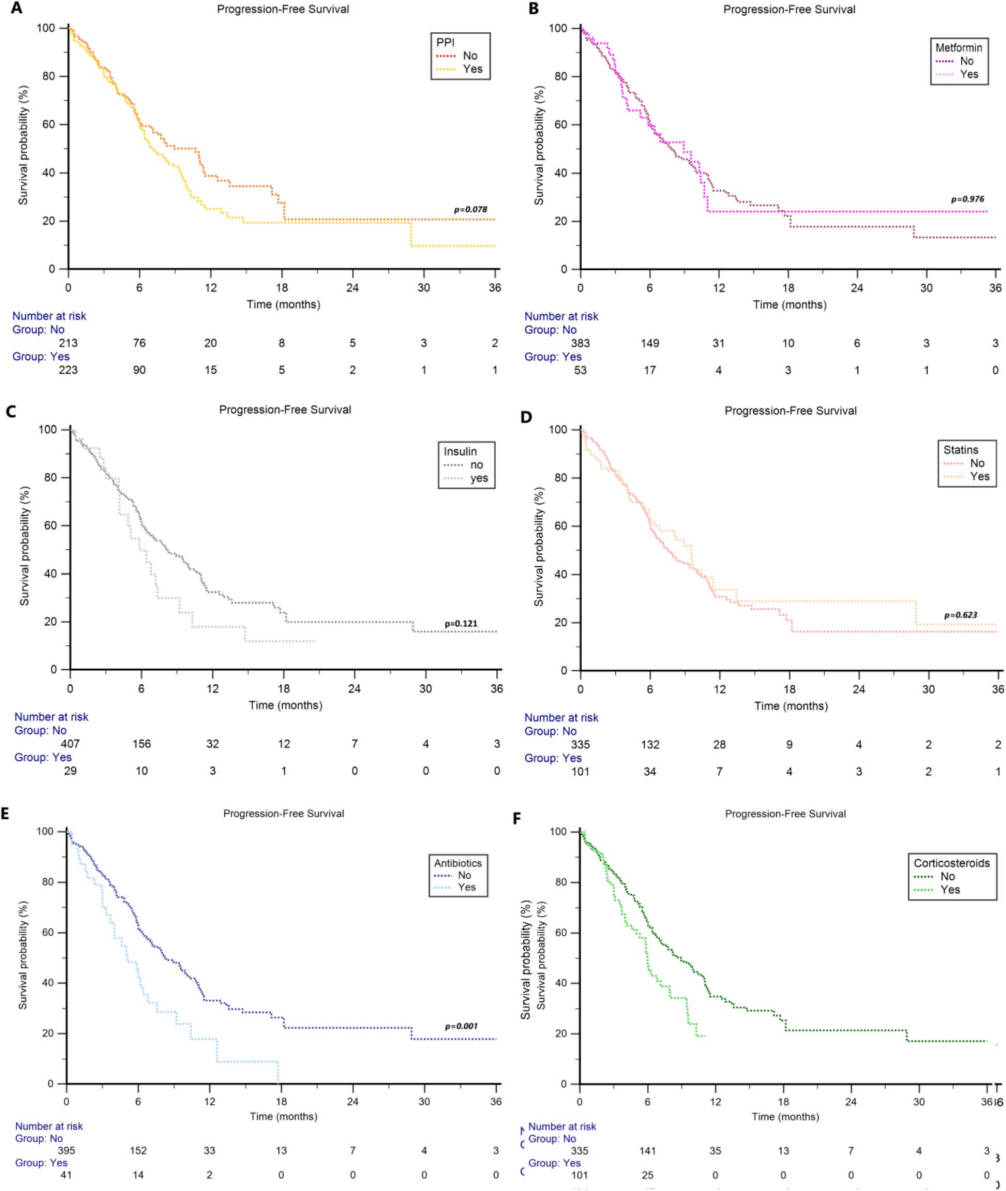
Progression-Free Survival in patients treated with Enfortumab Vedotin (EV) stratified by concomitant medications

The median OS in the overall study population was 13.4 months (95%CI 11.3 − 47.3). ATB users, compared to non-users, showed significant shorter median OS (7.3 months, 95%CI 5.0 − 12.3 vs 13.7 months, 95%CI 12.2 − 47.3, *p* = 0.001, Fig. 1E), with a 1y-OS rate of 33% vs 58% (*p* < 0.001). Shorter median OS was also observed in corticosteroid users compared to non-users (8.4 months, 95%CI 6.6 − 10.0 vs 14.2 months, 95%CI 12.7 − 47.3, *p* < 0.001, Fig. 1F), with a 1y-OS rate of 36% vs 61% (*p* < 0.001). Otherwise, concomitant use of PPI (users: 12.3 months, 95%CI 9.7 − 47.3 vs non-users: 15.1 months, 95%CI 12.2 − 20.0 *p* = 0.343, Fig. 1A), metformin (users: 11.1 months, 95%CI 7.2 − 18.7 vs non-users: 13.6 months, 95%CI 11.9 − 47.3 *p* = 0.665, Fig. 1B), insulin (users: 9.2 months, 95%CI 5.4 − 14.7 vs non-users: 13.7 months, 95%CI 11.9 − 47.3 *p* = 0.223, Fig. 1C), or statins (users: 13.7 months, 95%CI 8.9 − 35.4 vs non-users: 13.4 months, 95%CI 11.1 − 47.3 *p* = 0.702, Fig. 1D) were not significantly associated with OS. The median PFS in the overall study population was 8.0 months (95%CI 6.8 − 47.3). Concomitant use of ATBs (5.1 months 95%CI: 3.3 − 17.7 vs 8.3 months, 95%CI 7.1 − 47.3, *p* = 0.001, Fig. 2E) or corticosteroids (6.0 months 95%CI 4.6 − 7.9 vs 8.9 months, 95%CI 7.2 − 47.3, *p* = 0.004, Fig. 2F) was significantly associated with shorter PFS (Fig. 1), in contrast to the data on the concomitant use of PPIs (users: 7.1 months, 95%CI 6.3 − 47.3 vs non-users: 10.7 months, 95%CI 7.1 − 11.4, *p* = 0.078, Fig. 2A), metformin (users: 8.9 months, 95%CI 4.1 − 10.7 vs non-users: 7.9 months, 95%CI 6.7 − 47.3, *p* = 0.976, Fig. 2B), insulin (users: 5.9 months, 95%CI 4.1 − 7.4 vs non-users: 8.2 months, 95%CI 6.9 − 47.3, *p* = 0.121, Fig. 2C) or statins (users: 9.5 months, 95%CI 6.0 − 11.4 vs non-users: 7.6 months, 95%CI 6.4 − 47.3, *p* = 0.623, Fig. 2D).

### Objective response rate

**Fig. 3:**
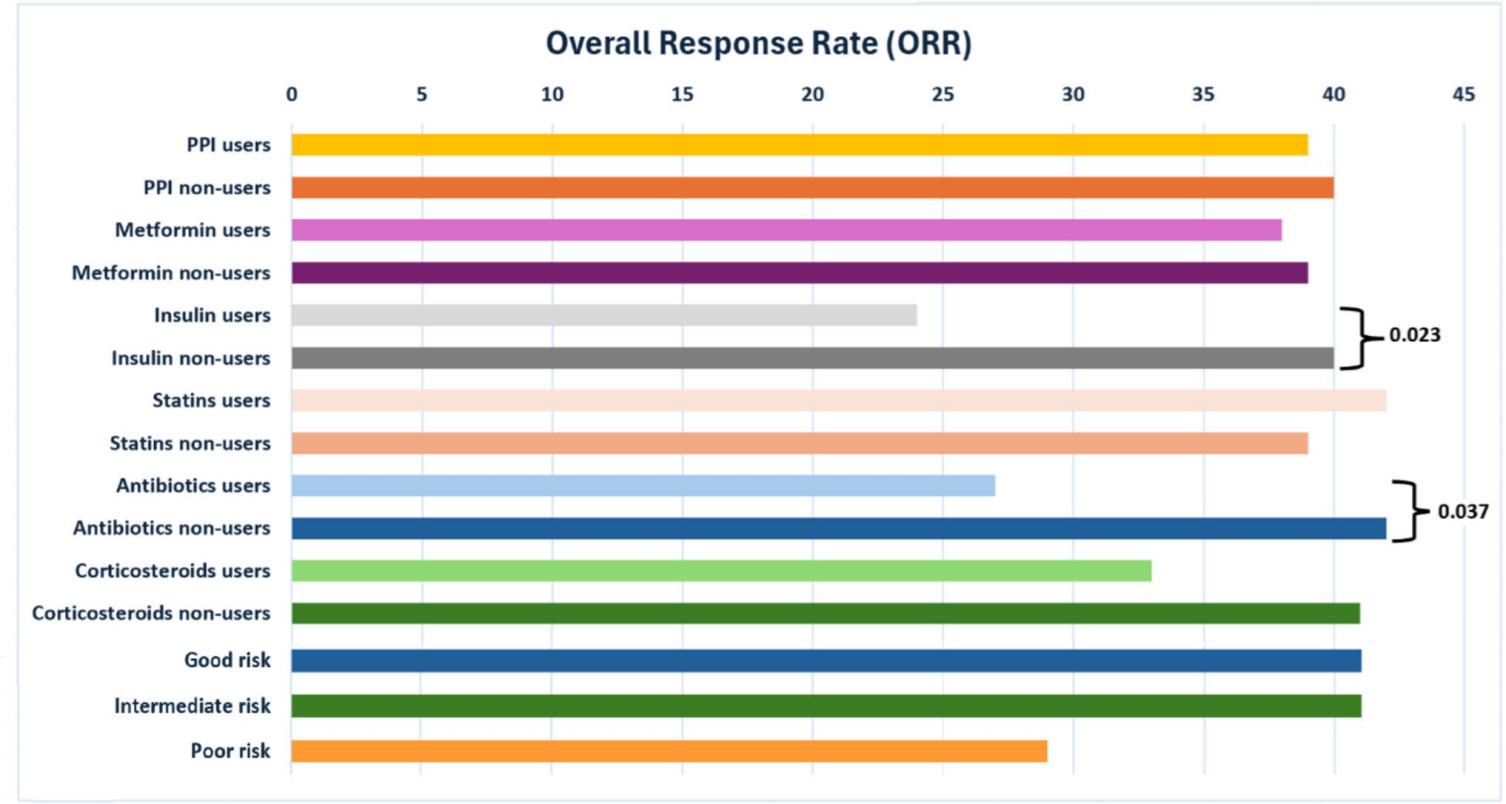
Overall Response Rate (ORR) in patients treated with enfortumab vedotin stratified by concomitant medications. Significant p values calculated by Fisher’s exact text were reported in bold

We further analyzed the relationship between concomitant medications and response to EV in terms of ORR. In the overall study population we reported an ORR of 39%. In Fig. 3 we showed the ORR stratified by concomitant medications. We observed a significant difference in the ORR of insulin non-users vs users (+ 16%) and of antibiotics non-users vs users (+ 15%), while corticosteroids non-users showed a not statistically significant + 8% compared to users (Fig. 3).

### Outcomes according to concomitant drug score

**Fig. 4:**
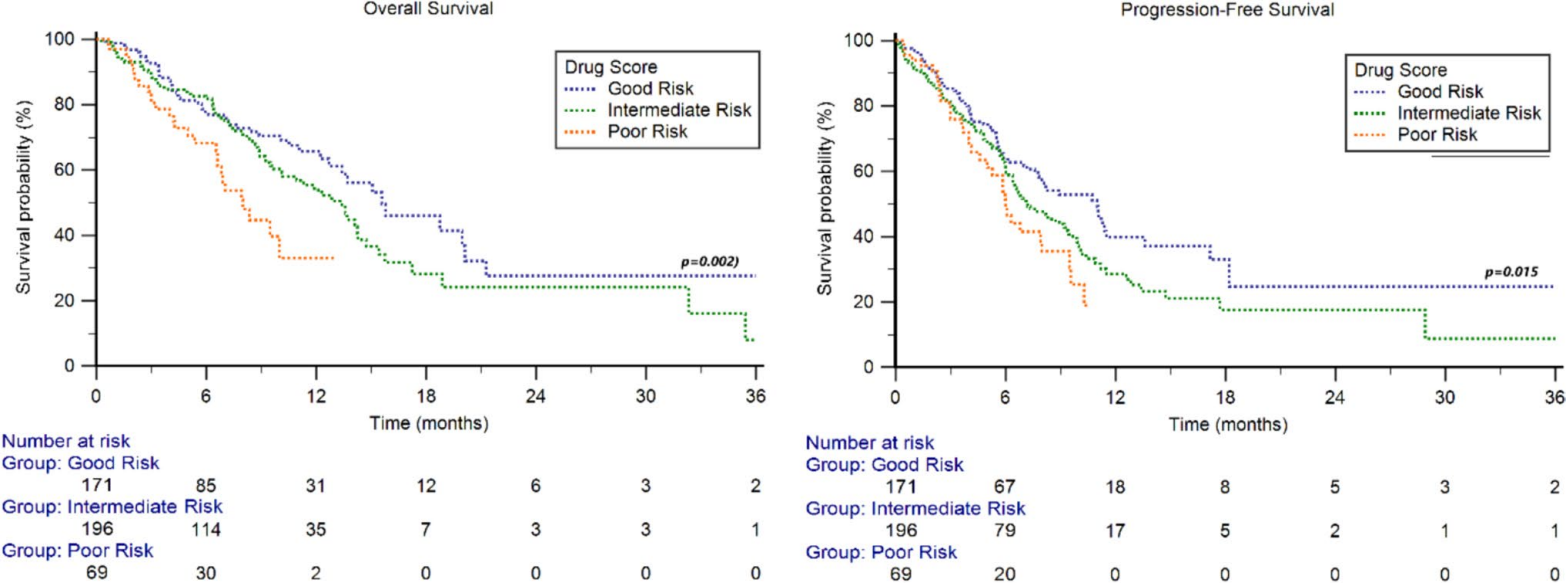
Overall Survival and Progression-Free Survival stratified by concomitant drug score

Patients were stratified into three groups based on their concomitant drug score as described above: good risk (n = 171), intermediate risk (n = 196), and poor risk (n = 69). The median OS was 15.6 months (95%CI 12.7–20.1) for the good risk group, 13.4 months (95%CI 10.2–47.3) for the intermediate risk group, and 8.0 months (95%CI 6.6–9.9) for the poor risk group, (p = 0.002). The median PFS was 11.0 months (95%CI 7.8–13.6) for the good risk group, 7.2 months (95%CI 6.4–47.3) for the intermediate risk group and 6.0 months (95%CI 4.6–8.0) for the poor risk group (p = 0.015) (Fig. 4).

In the Cox multivariate analysis, the concomitant drug score remains a significant factor predicting both OS (HR = 1.32 [95% CI 1.03−1.68], p = 0.026) and PFS (HR = 1.23 [95% CI 1.01 − 1.51], p = 0.044). Other independent prognostic factor for OS and PFS identified in the multivariate analysis was ECOG-PS. The results of the univariate and multivariate Cox analysis are summarized in Table 2.

**Table 2:** Univariate and multivariate Cox analyses

Overall survival	Univariate cox Regression		Multivariate cox Regression	
	HR (95%CI)	*p-value*	HR (95%CI)	*p-value*
Sex (females vs males)	1.07 (0.74 − 1.56)	0.709		
Age (≥ 70y vs < 70y)	0.79 (0.57 − 1.08)	0.139		
ECOG-PS (≥ 2 vs 0–1)	3.77 (2.61 − 5.44)	** < 0.001**	3.14 (2.10 − 4.69)	** < 0.001**
Smokers vs no-smokers	1.11 (0.79 − 1.58)	0.524		
Histology (mixed vs pure UC)	0.85 (0.53 − 1.35)	0.483		
Upper vs Lower urinary tract	0.74 (0.52 − 1.06)	0.102		
Synchronous metastatic disease (yes vs no)	1.07 (0.77 − 1.49)	0.687		
Lymph node metastases (yes vs no)	1.29 (1.06 − 1.57)	**0.011**	1.19 (0.98 − 1.43)	0.069
Lung metastases (yes vs no)	1.23 (0.90 − 1.68)	0.199		
Bone metastases (yes vs no)	1.42 (1.03 − 1.95)	**0.031**	1.20 (0.87 − 1.66)	0.277
Liver metastases (yes vs no)	1.51 (1.09 − 2.10)	**0.012**	1.10 (0.78 − 1.56)	0.569
Concomitant Drug Score (3–4 vs 1–2 vs 0)	1.49 (1.18 − 1.89)	** < 0.001**	1.32 (1.03 − 1.68)	**0.026**

Progression-free survival	Univariate cox regression		Multivariate cox regression	
	HR (95%CI)	*p-value*		HR (95%CI)
Gender (females vs males)	1.14 (0.84 − 1.55)	0.404		
Age (≥ 70y vs < 70y)	0.90 (0.67 − 1.17)	0.404		
ECOG-PS (≥ 2 vs 0–1)	2.48 (1.78 − 3.46)	** < 0.001**	2.17 (1.51 − 3.10)	** < 0.001**
Smokers vs no-smokers	1.12 (0.83 − 1.52)	0.454		
Histology (mixed vs pure UC)	0.85 (0.53 − 1.35)	0.483		
Upper vs Lower urinary tract	0.82 (0.60 − 1.16)	0.207		
Synchronous metastatic disease (yes vs no)	1.16 (0.88 − 1.54)	0.292		
Lymph node metastases (yes vs no)	1.20 (0.99 − 1.45)	0.052		
Lung metastases (yes vs no)	1.03 (0.78 − 1.36)	0.826		
Bone metastases (yes vs no)	1.31 (0.99 − 1.74)	0.060		
Liver metastases (yes vs no)	1.45 (1.09 − 1.92)	**0.011**	1.15 (0.86 − 1.55)	0.153
Concomitant Drug Score (3–4 vs 1–2 vs 0)	1.35 (1.10 − 1.65)	**0.004**	1.23 (1.01 − 1.51)	**0.044**

Statistically significant differences are in bold

*ECOG-PS* Eastern cooperative oncology group-performance status, *UC* Urothelial carcinoma

The ORR was 41% in both good and intermediate risk groups and 29% in the poor risk group (*p* = 0.128).

## Discussion

The role of concomitant medication and its potential impact on the outcome of cancer patients receiving systemic therapy has become a subject of increasing interest. As an ADC therapeutic, the potential of direct drug-drug interactions in EV are limited according to the findings of prior pharmacological studies [21]. However, data on potential impact of concomitant medication on the outcomes of mUC patients treated with EV in a real-life clinical setting are missing. The results of the present study indicate a negative impact of concomitant use of ATBs and corticosteroids on the outcome of mUC patients receiving EV after platinum-based chemotherapy followed by second-line pembrolizumab or avelumab maintenance. Moreover, these findings illustrate the prognostic utility of the concomitant drug score, originally developed for patients undergoing immunotherapy. Its impact on patient survival outcomes was confirmed in the multivariate analysis.

Although ATBs, PPIs, corticosteroids, and antidiabetics are widely prescribed in the global population, the potential impact of these drugs on the outcome of mUC patients receiving EV is unknown. It is well established that the gut microbiota plays a key role in the anti-tumor immune response, which in turn impacts cancer development, progression, and treatment outcomes [22, 23]. Additionally, gut microbiota can modulate the efficacy of anti-cancer therapies [22–24]. A growing body of evidence suggests that several concomitant medications are able to influence the outcomes of cancer patients by modulating the composition and function of gut microbiota. Although this phenomenon has recently been investigated primarily in patients treated with immunotherapy, it is likely not exclusive to this treatment modality. Numerous studies have confirmed that the ATB use has a tremendous impact on the composition and functionality of the gut microbiota by reducing its overall diversity [25, 26]. The results of a post hoc analysis of IMvigor210 and IMvigor211 show that ATB use was associated with worse OS (HR: 1.44, 95% CI 1.19–1.73) and PFS (HR: 1.24, 95% CI 1.05–1.46) in patients receiving atezolizumab [8]. The results of the present study indicate that ATB use is associated with inferior survival and ORR among mUC patients treated with EV. This suggests that the use of ATBs may have implications for the efficacy of ADC therapies, similar to their known impact on ICIs. ATBs can disrupt the gut microbiota, which plays a critical role in modulating the immune system and maintaining an immune-activated tumor microenvironment. This disruption may impair immune-mediated anti-tumor responses, which could potentially influence the efficacy of ADCs. While the mechanisms of ADCs primarily involve targeted cytotoxicity, their interactions with the immune system may still render them susceptible to microbiota-mediated effects. Future studies are needed to investigate whether ATB-induced alterations in gut microbiota could directly affect ADC outcomes, as has been observed with ICIs. It is worth noting that the use of ATBs in mUC patients is quite frequent, especially because they often have uretero-cutaneous stomas, uretero-ileocutaneous stomas, or nephrostomies, or have urinary stents [27, 28]. These are all conditions that predispose to infection and the use of ATBs. PPIs represent another type of drugs with significant impact on gut microbiota including changes in composition and reduction of diversity [29–31]. Additionally, PPIs can influence the gut microbiota through induction of hormonal changes and impaired nutrient absorption [32]. The association between concomitant use of PPIs and inferior survival outcomes has been recently reported in several retrospective studies focusing on mUC patients receiving ICIs [8–12]. Our findings indicate that the use of PPI does not have a significant impact on the outcomes of patients treated with EV. Although the association between PPI use and survival did not reach statistical significance in the present study, a detectable trend towards inferior survival was observed in PPI users. This aligns with findings from previous research suggesting that PPIs, through their impact on gut microbiota may influence immune responses and the efficacy of anticancer therapies. While our study cannot confirm a direct relationship due to the lack of statistical significance and potential confounding factors, this trend highlights the need for further investigation into the role of PPIs in modulating treatment outcomes. Corticosteroids are drugs with complex effects potentially impairing anti-tumor surveillance. The negative impact of concomitant corticosteroid use has been the subject of extensive recent investigation, particularly in cancer patients undergoing immunotherapy with ICIs. They have well-known immunosuppressive activity and, moreover, they also impact the gut microbiota by affecting mucus production and secretion [33]. It has been proposed that corticosteroid use is associated with decreased survival outcomes in cancer patients treated with ICIs [13, 14]. In the present study, we observed inferior survival outcomes for mUC patients receiving EV which is in line with data reported in mUC patients treated with ICIs. Despite the lack of data on specific indications for the use of corticosteroids in the present study population, we speculate that the most likely indication for their use was the management of disease-related symptoms, such as asthenia, poor appetite, dyspnea, pain, etc. [34].

In light of the above findings, a comprehensive drug score was developed to provide prognostic information regarding concomitant medication with ATBs, PPIs and corticosteroids [15]. It has been validated on several cancer types including non-small cell lung cancer, renal cell carcinoma and UC, in patients treated with ICIs [17–19]. The prognostic capability of the drug score has recently been successfully validated by Taguchi et al. in a retrospective study including 242 patients with la/mUC treated with third-line pembrolizumab [19]. The results are in agreement with our findings, although our study assessed patients treated with ADC therapy instead of ICI. It is worth mentioning that because the gut microbiota can significantly influence the efficacy of anticancer therapies, there is a need for studies of the microbiota along with concomitant medications that could provide a stronger basis for understanding the observed associations and mechanisms behind the impact of concomitant medications on treatment outcomes.

The potential anticancer properties of statins and metformin have been postulated in several experimental studies [35–39]. Nevertheless, the available clinical data from patients with UC is inconclusive, and further investigation is required to elucidate the efficacy of these agents in this context [12, 40, 41]. The findings of the present investigation suggest that there is no significant correlation between patient outcomes and the concomitant use of statins or metformin. The use of insulin or insulin analogs has been reported in association with increased cancer risk compared to other antihyperglycemic drugs [42]. On the other hand, their impact on the prognosis of cancer patients remains poorly understood. Nevertheless, the available data are inconclusive, with several findings indicating that insulin may have an adverse effect on the clinical course, resulting in a shorter cancer-specific survival [43–45]. We observed significantly lower ORR for insulin users. In the survival outcomes, a trend towards inferior outcomes was visible among insulin users; however, the results did not reach statistical significance. These results should be interpreted with caution and further validation is needed.

The present study is characterized by a number of strengths and limitations. To the best of our knowledge, this is the first study exploring the impact of concomitant medication on the effectiveness of EV monotherapy in mUC patients. The study population is large, including patients from various countries, and represents a truly global patient dataset. The limitations are primarily due to the retrospective design. The endpoints were measured locally by investigators (without centralized review), and we only have data on drug classes, not individual molecules. Additionally, we lack information regarding the dose and duration of concomitant treatments, as well as indications for their use, and the burden of comorbidities in the analysed patient cohort. The association of medications with adverse outcomes may be results of an indication bias, i.e. the need to administer a given treatment may be the factor explaining poor outcome rather than the effect of the medication itself. Also, both ATB and corticosteroid use were associated with ECOG-PS > 2, suggesting that patients with worse performance status were more likely to receive these medications. In the present analysis, we attempted to account for this by including ECOG-PS as a variable in the multivariate model. While this helps to adjust for the effect of performance status, we recognize that residual confounding cannot be completely excluded, particularly in a retrospective study where unmeasured factors may influence outcomes. All of the above limitations indicate that the results of the present study should be interpreted with caution and that future studies need to prospectively collect more detailed data, including the timing, dose, and duration of concomitant medications, to better understand their influence on treatment outcomes.

## Conclusions

The findings of the present real-world retrospective study suggest an association of concomitant use of ATBs and corticosteroids and inferior survival outcomes in patients with previously treated mUC receiving EV. The prognostic utility of the concomitant drug score was successfully validated, indicating that it is a reliable prognostic tool for mUC patients treated with EV, not limited to immunotherapy, where it was originally developed. As the first study to evaluate the impact of concomitant medication in mUC patients receiving EV, further research in the field remains warranted.

## Data Availability

No datasets were generated or analysed during the current study.

## Code availability

Not applicable’ for that section.
